# The Author of Thesaurus

**Published:** 1869-11

**Authors:** 


The Author of Thesaurus.—Dr. Peter Mark Roget, who died in England on Friday, at the age of 90 years, was a Swiss by origin, and his mother was a sister of Sir Samuel Romilly. He stood high as a physician, but is best known by his valuable "Thesaurus of English Words and Phrases," and his contribution to the Bridgewater Treatises on "Animal and Vegetable Physiology. He wrote various mathematical papers, and contributed largely to the medical and philosophical reviews. His great work of English words and phrases was revised and enlarged by Barnas Sears in 1854, while he was Secretary of the Massachusetts Board of Education, and published by Gould & Lincoln. It has passed through several editions since, and though it was the first work of its kind, the completeness of its plan and its fullness of details left little to be supplied.—*Boston Evening Transcript.*

